# Falls Are Associated With Decreased Autonomy, and Self-Efficacy Moderates This Relation: Results From a National Study

**DOI:** 10.3389/fpsyt.2019.00447

**Published:** 2019-06-21

**Authors:** André Hajek, Hans-Helmut König

**Affiliations:** Department of Health Economics and Health Services Research, Hamburg Center for Health Economics, University Medical Center Hamburg-Eppendorf, Hamburg, Germany

**Keywords:** falls, autonomy, self-efficacy, cross-sectional study, cohort study

## Abstract

The first aim of this study was to examine the association between falls and perceived autonomy. The second aim was to investigate whether this association is moderated by self-efficacy. Cross-sectional data were drawn from the German Ageing Survey—a nationally representative sample of individuals living in private households aged 40 and above (*n* = 7,746) in Germany. Perceived autonomy was quantified according to Schwarzer. Self-efficacy was assessed using a widely established scale by Schwarzer and Jerusalem. With covariates being adjusted, linear regressions revealed that experiencing a fall in the past 12 months was associated with lower perceived autonomy (β = −.09, *p* < .001). General self-efficacy moderated this association (β = .08, *p* = .02). Findings emphasized the association between falls and perceived autonomy as well as the moderating role of self-efficacy. Future longitudinal studies are required to gain insights into the temporal relationship between these variables.

## Introduction

Experiencing a fall is a frequent phenomenon among individuals aged 65 years and over (1), and the proportion of individuals who experience a fall increases with age (2). Because it is presumed that the number of individuals aged 65 years and over will increase significantly due to demographic changes, and because falls are associated with negative health outcomes, various studies have examined the correlates of falls (3).

Falls can lead to increased depressive symptoms or reduced physical activity (4, 5). It has also been shown that falls are associated with loneliness or decreased subjective well-being (6, 7). However, it is not known whether falls are associated with perceived autonomy, which is a fundamental and general psychological need (8). Ryan and Deci (8) define autonomy as the “experience of choice”. Perceived autonomy leads, for example, to greater psychological well-being (9) and is related to mindfulness (10). Roe et al. (11) found that individuals who did not reflect on previous falls, or did not know why they fell, restricted their lives. However, this study did not explicitly focus on the association between falls and perceived autonomy.

With data drawn from a nationally representative sample of community-dwelling adults aged 40 and older, the aims of this study were

1. to investigate the association between falls and perceived autonomy and
2. to investigate whether the association between falling and perceived autonomy is moderated by self-efficacy.

We hypothesized that falls are associated with a decreased perceived autonomy. Individuals experiencing a fall in the past year may have a lower perceived freedom. More specifically, individuals who experienced a fall may avoid activities of daily living and may avoid leaving the house because they were afraid of falling again (12).

General self-efficacy, the belief in one’s capabilities to accomplish tasks or achieve goals, may act as a moderating factor in the relation between falling and perceived autonomy. It appears plausible that individuals who score high in self-efficacy may believe in their ability to achieve goals (e.g., the ability to live autonomously). Thus, self-efficacy might moderate the relationship between falls and perceived autonomy. Or to put it another way: We hypothesized that self-efficacy can moderate the link between falls and autonomy. This is also supported by other empirical studies showing that self-efficacy can act as a coping mechanism [e.g., to buffer against negative effects of informal caregiving (13)]. Furthermore, it has been shown that there is a significant association between falls and (fall-related) self-efficacy (14). However, there is a lack of studies showing that self-efficacy can act as moderating factor in the link between falls and outcome measures such as autonomy.

## Methods

### Sample

We used data from the German Ageing Survey (“Deutscher Alterssurvey,” DEAS). The DEAS study is a longitudinal cohort-based survey of individuals living in private households aged 40 and over (individuals in the second half of life) in Germany.

The current study used the data from the fifth wave (year 2014) because this wave includes measures of autonomy and falls (*n* = 7,746 individuals provided data on experiencing a fall and perceived autonomy). Neither variable was measured in previous waves. In 2014, the response rate was approximately 61% (panel sample) and about 25% (cross-sectional sample). Neller (15) showed that the response rate is similar to that of other German survey studies. Furthermore, the participation rate mirrors the trend of declining participation rates in Germany. The DEAS study is described in further detail elsewhere (16).

### Ethical Statement

Written informed consent was provided by all study participants. The DEAS study follows the principles of the Declaration of Helsinki.

An ethical statement for the DEAS study was deemed unnecessary. Prior to each wave of data collection, the permanent advisory board for the study receives detailed information about the sampling method, the instruments used in the DEAS study, and the consent among respondents to participate. The permanent advisory board concluded that the DEAS study did not require approval from an ethics committee.

### Dependent Variables

Perceived autonomy was quantified using a scale developed by Schwarzer (17). It consists of four items (index score from 1 to 4). Items are as follows: “In my daily life, I get along well on my own,” “I make my own decisions and don’t allow others to protect me,” “I organize my life according to my own ideas,” and “I cope with my daily life without outside help”. The higher the index score, the higher perceived autonomy (Cronbach’s alpha = .81).

### Independent Variables

The experience of a fall in the past 12 months (yes; no) was measured. This is a common way to measure the history of falls (18, 19). Schwarzer and Jerusalem (20, 21) developed a scale to quantify self-efficacy. The original scale consisted of 10 items. The short scale used in this study comprised five items (with four levels each). The short scale was constructed by members of the DEAS study in direct consultation with Ralf Schwarzer. The psychometric properties of the scale have been demonstrated elsewhere (22).

Based on empirical evidence and theoretical considerations, covariates were included. Thus, socioeconomic variables were included in the regression model: sex, age, civil status (single; divorced; widowed; married, living separated from spouse; married, living together with spouse) as well as individual monthly net equivalent income [Organisation for economic co-operation and development (OECD) scale]. With respect to lifestyle variables, the frequency of sports activities and the frequency of alcohol consumptions was recorded (both: “never,” “rarer than once a month,” “one to three times a month,” “once a week,” “several times a week,” and “daily”). Moreover, smoking status was included (daily smoker; casual smoker; former smoker; non-smoker).

Depressive symptoms (15-item version of the Center for Epidemiological Studies Depression Scale, CES-D (23; ranging from 0 to 45; with higher values reflecting more depressive symptoms) and the number of physical illnesses (cardiac and circulatory disorders; bad circulation; joint, bone, spinal, or back problems; respiratory problems, asthma, or shortness of breath; stomach and intestinal problems; cancer; diabetes; gall bladder, liver, or kidney problems; bladder problems; eye problems, vision impairment; ear problems, hearing problems; and other illnesses or health problems) were also included as covariates. In addition, physical functioning was assessed by means of the subscale “physical functioning” of the SF-36 (ranging from 0 = worst score to 100 = best score) (24). Furthermore, self-rated health from 1 = “very good” to 5 = “very bad”) served as predicting variable.

### Statistical Analysis

Sample characteristics were computed, and pairwise correlations were calculated. With covariates being adjusted, multiple linear regressions were performed to test the relationship between falls in the past 12 months and perceived autonomy and to test whether self-efficacy moderates this relationship. Statistical significance was deemed when the *p*-value was less than .05. Stata 15.0 (Stata Corp, College Station, TX, USA) was used to conduct statistical analysis.

### Data Availability

The data used in this study are third-party data. The anonymized data sets of the DEAS (1996, 2002, 2008, 2011, and 2014) are available for secondary analysis. The data were made available to scientists at universities and research institutes for scientific purposes. The use of the data is subject to written data protection agreements. Microdata (referring to individuals) of all the completed waves of the German Ageing Survey (DEAS) is available free of charge to scientific researchers for non-profitable purposes. The Research Data Centre of the DZA (FDZ-DZA) provides access and support to scholars interested in using DEAS for their research. However, for reasons of data protection, signing a data distribution contract is required before data can be obtained. Please see for further information (data distribution contract) https://www.dza.de/en/fdz/german-ageing-survey/access-to-deas-data.html.

## Results

### Description of the Sample and Bivariate Correlations

Sample characteristics for individuals providing data for experiencing a fall and perceived autonomy are summarized in **Table 1**. In sum, 1,355 (17.5%) experienced a fall in the past year. Perceived autonomy was on average 3.5 (± 0.5), and mean self-efficacy was 3.1 (± 0.4).

**Table 1:** Sample characteristics (German Ageing Survey, fifth wave, *n* = 7,746).

	*N/Mean*	%/(SD)
Gender: Female	3,936	50.8%
Age in years	64.5	11.2
Marital status: married and living together with spouse	5,422	70.1%
Monthly net equivalent income in Euro	1946.0	1380.6
Body mass index (BMI)	26.9	4.6
Smoking status: - Daily	1,070	13.9%
- Yes, sometimes	305	4.0%
- Not anymore	2,865	37.1%
- Never been smoker	3,476	45.0%
Consumption of alcohol: - Daily	932	12.0%
- Several times a week	1,886	24.4%
- Once a week	1,235	16.0%
- One to three times a month	944	12.2%
- Less frequently	1,860	24.0%
- Never	878	11.4%
Physical activity: - Daily	645	8.3%
- Several times a week	2,111	27.3%
- Once a week	1,420	18.3%
- One to three times a month	579	7.5%
- Less frequently	909	11.7%
- Never	2,081	26.9%
Self-rated health (from 1 = ”very good” to 5 = ”very bad”)	2.5	0.8
Number of physical illnesses (from 0 to 11)	2.6	1.9
Depressive symptoms (ranging from 0 = no depressive symptoms to 45 = severe depressive symptoms)	6.6	6.0
Physical functioning (from 0 = lowest score to 100 = best score)	81.7	22.9
Experiencing a fall in the preceding 12 months	1,355	17.5%
Perceived autonomy (from 1 = low perceived autonomy to 4 = high perceived autonomy)	3.5	0.5
Self-efficacy (from 1 = low self-efficacy to 4 = high self-efficacy)	3.1	0.4

Beta coefficients are reported. Cluster-robust standard errors are in parentheses. ***p < .001, **p < .01, *p < .05, + p < .10. Perceived autonomy was quantified using a scale developed by Schwarzer, ranging from 1 to 4. Self-efficacy was quantified using a scale developed by Schwarzer and Jerusalem, ranging from 1 to 4. Depressive symptoms were quantified using the CES-D, ranging from 0 to 45. Physical functioning was quantified using the subscale “physical functioning” of the SF-36, ranging from 0 to 100.

Pairwise correlations (with Bonferroni-adjusted significance levels) revealed that falls were negatively associated with perceived autonomy (*r* = −.13, *p* < .001) and self-efficacy (*r* = −.10, *p* < .001) (further details are not shown but available upon request).

### Regression Analysis

With several covariates being adjusted (**Table 2**), the association between falls and perceived autonomy was examined (first model: first column). This model was extended by adding self-efficacy (second column). Furthermore, an interaction term (falls × self-efficacy) was added (third column).

**Table 2:** Determinants of perceived autonomy. Results of multiple linear regression analysis (German Ageing Survey, fifth wave).

Independent variables	Perceived autonomy	Perceived autonomy	Perceived autonomy
Gender: female (Ref.: male)	0.143***	0.136***	0.136***
	(0.013)	(0.012)	(0.012)
Age in years	0.002*	0.002**	0.001**
	(0.001)	(0.001)	(0.001)
Marital status: other marital statuses (Ref.: married and living together with spouse)	0.166***	0.163***	0.164***
	(0.012)	(0.012)	(0.012)
Monthly net equivalent income in Euro	0.000***	0.000**	0.000**
	(0.000)	(0.000)	(0.000)
Body mass index (BMI)	0.005***	0.003*	0.003*
	(0.001)	(0.001)	(0.001)
Smoking status: - Yes, sometimes (Ref.: daily)	0.025	0.018	0.018
	(0.033)	(0.030)	(0.030)
- Not anymore	−0.030	−0.022	−0.022
	(0.019)	(0.018)	(0.018)
- Never been a smoker	−0.046*	−0.033+	−0.033+
	(0.019)	(0.018)	(0.018)
Consumption of alcohol: - Several times a week (Ref.: daily)	−0.017	−0.019	−0.019
	(0.020)	(0.019)	(0.019)
- Once a week	0.001	−0.008	−0.008
	(0.023)	(0.021)	(0.021)
- One to three times a month	−0.002	−0.006	−0.007
	(0.024)	(0.022)	(0.022)
- Less frequently	0.030	0.022	0.022
	(0.021)	(0.020)	(0.020)
- Never	0.025	0.014	0.015
	(0.026)	(0.025)	(0.025)
Physical activity: - Several times a week (Ref.: daily)	0.008	0.010	0.011
	(0.022)	(0.021)	(0.021)
- Once a week	−0.022	−0.011	−0.009
	(0.024)	(0.022)	(0.022)
- One to three times a month	−0.006	−0.006	−0.006
	(0.029)	(0.028)	(0.028)
- Less frequently	−0.020	−0.016	−0.015
	(0.026)	(0.025)	(0.025)
- Never	−0.024	−0.021	−0.021
	(0.024)	(0.022)	(0.022)
Self-rated health (from 1 = “very good” to 5 = ”very bad”)	−0.020*	−0.007	−0.007
	(0.009)	(0.009)	(0.009)
Number of physical illnesses (from 0 to 11)	−0.015***	−0.007+	−0.007+
	(0.004)	(0.004)	(0.004)
Depressive symptoms (ranging from 0 = no depressive symptoms to 45 = severe depressive symptoms)	−0.010***	−0.003**	−0.003**
	(0.001)	(0.001)	(0.001)
Physical functioning (from 0 = lowest score to 100 = best score)	0.005***	0.004***	0.004***
	(0.000)	(0.000)	(0.000)
Fall in the preceding 12 months (Ref.: no)	−0.086***	−0.078***	−0.312**
	(0.016)	(0.016)	(0.105)
Self-efficacy (from 1 = low self-efficacy to 4 = high self-efficacy)		0.394***	0.381***
		(0.014)	(0.015)
Interaction term: Fall × self-efficacy			0.078*
			(0.033)
Constant	3.931***	2.712***	2.764***
	(0.086)	(0.091)	(0.094)
			
Observations	6,930	6,911	6,911
*R* ^2^	.148	.244	.245

Beta coefficients are reported. Cluster-robust standard errors are in parentheses. ***p < .001, **p < .01, *p < .05, + p < .10. Perceived autonomy was quantified using a scale developed by Schwarzer, ranging from 1 = low autonomy to 4 = high autonomy. Self-efficacy was quantified using a scale developed by Schwarzer and Jerusalem, ranging from 1 to 4. Depressive symptoms were quantified using the CES-D, ranging from 0 to 45. Physical functioning was quantified using the subscale “physical functioning” of the SF-36, ranging from 0 to 100.

In the first model, falls were negatively associated with perceived autonomy (β = −.09, *p* < .001). In the second model, self-efficacy was positively associated with perceived autonomy (β = .39, *p* < .001). The interaction term (β = .08, *p* = .02) achieved statistical significance. In other words, the association between falls and autonomy is less pronounced when self-efficacy is high.

As for the covariates (first column), perceived autonomy was positively associated with being female (β = .14, *p* < .001), higher age (β = .002, *p* < .05), being married (β = .17, *p* < .001), higher income (β = .000, *p* < .001), higher BMI (β = .01, *p* < .001), and higher physical functioning (β = .01, *p* < .001). Moreover, the outcome measure was negatively associated with poorer self-rated health (β = −.02, *p* < .05), more depressive symptoms (β = −.01, *p* < .001), and increases in the number of physical illnesses (β = −.02, *p* < .001).

## Discussion

With data drawn from a nationally representative sample of community-dwelling adults in the second half of life, the purpose of the current study was to investigate the relation between falls and perceived autonomy. Following adjustment for covariates, multiple linear regressions revealed that experiencing a fall in the past 12 months was associated with lower perceived autonomy. This association was moderated by self-efficacy.

We are not aware of any published quantitative research examining the association between falls and perceived autonomy. Moreover, no research has been conducted to date on the moderating role of self-efficacy in the association between falls and perceived autonomy.

The association between falls and decreased perceived autonomy may be explained by the fact that those who have experienced a fall tend to avoid daily activities, such as meeting with friends, because they may fear falling again (12). However, it has been shown that maintaining autonomy can offset the risk of falls among community-dwelling individuals aged 65 or older (25). In practice, this might not always be the case. Future research based on longitudinal data is necessary to clarify the relationship between falls and autonomy.

In our study, it has additionally been shown that the association between falls and autonomy is less pronounced when self-efficacy is high. We assume that individuals who score high in self-efficacy may believe in his or her own abilities to reach the goal of living autonomously. Thus, it appears plausible that self-efficacy moderates this relationship.

The present study has some strengths. To the best of our knowledge, this is the first quantitative study that examines the link between falls and perceived autonomy. Data were drawn from a nationally representative sample of individuals living in private households in the second half of life. Perceived autonomy and general self-efficacy were measured using scales with established psychometric properties. However, some limitations are also worth noting. It has been shown that a small sample selection bias exists in the German Ageing Survey (26). In addition, further information on falls (such as the number of falls or the severity) was not covered in the DEAS study. It is also worth highlighting that this is a cross-sectional study, restricting inference of causality. We cannot dismiss the possibility that our findings are biased by time-constant unobserved factors. Longitudinal studies are required to overcome this shortcoming. Other factors such as physical functioning or age can also moderate the link between falls and autonomy. Future studies are required to address this issue.

## Conclusion

In conclusion, findings emphasized the association between falls and perceived autonomy as well as the moderating role of self-efficacy in this relationship. Future longitudinal studies are required to gain insight into the temporal relationship between these variables.

## Author Contributions

AH and H-HK contributed to the design and concept of analyses, preparation of data, statistical analysis and interpretation of data, and preparation of the manuscript. Both authors critically reviewed the manuscript, provided significant editing of the article, and approved the final manuscript.

## Conflict of Interest Statement

The authors declare that the research was conducted in the absence of any commercial or financial relationships that could be construed as a potential conflict of interest.
